# MRgFUS for desmoid tumors within the thigh: early clinical experiences

**DOI:** 10.1186/s40349-017-0081-3

**Published:** 2017-02-03

**Authors:** Matthew D. Bucknor, Viola Rieke

**Affiliations:** Department of Radiology and Biomedical Imaging, University of California San Francisco, 185 Berry Street, Suite 350, San Francisco, CA 94107-5705 USA

**Keywords:** MRgFUS, Desmoid tumor, Case report, Ablation

## Abstract

**Background:**

Desmoid tumors are benign but locally aggressive non-malignant tumors derived from fibroblasts. Surgery, chemotherapy, and radiation therapy have been the mainstay of treatment, but recurrence is common and side effects can result in significant morbidity. In this case series, we highlight our experiences performing treatments in the thigh, including strategies for optimizing ablation size and safety.

**Case presentation:**

Since December 2014, 14 magnetic resonance-guided focused ultrasound (MRgFUS) treatments for desmoid tumors were performed at our institution in seven patients. Nine of these treatments were completed in three patients with large tumors within the posterior thigh.

The first was a 7-year-old boy who had previously been treated with surgical resection, intra-operative radiation, along with courses of vinblastine/methotrexate and sorafenib. Pretreatment tumor volume was 770 cm^3^ with 75% non-enhancing volume following the initial treatment. The first treatment was complicated by a third-degree far-field skin burn. Enhanced safety measures were developed to protect the far-field skin. The patient had four subsequent treatments over 14 months, without complication, with non-perfused volume of 85% on current imaging.

The second patient was a 21-year-old woman who had previously taken sulindac and celecoxib but had no other therapy. Pretreatment tumor volume was 740 cm^3^. The lateral decubitus position was used to minimize the amount of energy through the sciatic nerve. The first treatment resulted in a relatively low non-perfused volume of 30%. A follow-up treatment resulted in 75–80% ablation of the target.

The third patient was a 14-year-old girl with no prior treatment. Pretreatment tumor volume was approximately 440 cm^3^. The sciatic nerve was encased by the anteromedial portion of the mass. A lateral decubitus position and enhanced safety measures were again used. The first treatment resulted in a relatively low non-perfused volume of 30%, likely related to low energies. The second treatment resulted in 70–80% ablation.

**Conclusions:**

MRgFUS is an effective treatment for desmoid tumors of the thigh with a favorable side effect profile, allowing for repeated treatments if necessary. Ablation size and safety can be improved with far-field coupling devices, careful patient positioning, and optimized sonication planning.

## Background

Desmoid tumors, also known as aggressive fibromatosis, are monoclonal proliferations of fibroblasts arising in muscle and connective tissues. These tumors are rare, representing 0.03% of all neoplasms with an incidence of 2–4 million per year [1]. They most frequently occur sporadically in association with mutations of the beta-catenin gene, but a minority are associated with hereditary cancer syndromes, such as familial adenomatous polyposis. These tumors can be symptomatic or asymptomatic as a function of their local mass effect. Desmoid tumors also have variable clinical courses with growth, stability, or regression observed among different patients.

More established therapies for desmoid tumors include close observation, surgical resection, radiation therapy, and systemic medical therapy. Surgery, even with negative margins, can have recurrence rates as high as 50%, and multiple recurrences following surgery can result in local morbidity [2]. Radiation results in tumor control in approximately 72–76% of adults but is associated with pain, reduced motion, pathological fractures, and secondary malignancies [3]. Systemic medical therapies such as tamoxifen, nonsteroidal anti-inflammatory drugs, and cytotoxic chemotherapy demonstrate variable efficacy. More recently, cryoablation has been used successfully [4].

Magnetic resonance-guided focused ultrasound (MRgFUS) has emerged as a safe and effective treatment for control of desmoid tumors [5–7]. The thigh can be a challenging location where these tumors can become quite large, often in close proximity to neurovascular bundles such as the sciatic nerve, posing unique challenges and risks for treatment of patients with MRgFUS. In this case series, we highlight our experiences performing treatments in the thigh, including strategies for optimizing ablation size and safety.

## Case presentations

### Case 1

A 7-year-old boy with a history of a biopsy-proven sporadic desmoid tumor within the right anterolateral thigh was referred for MRgFUS therapy. The patient’s tumor was previously resected with intra-operative radiation therapy at the time of surgery (12 Gy). Initial recurrence was identified on imaging 10 months following the surgery. After the initial recurrence, the patient completed courses of vinblastine/methotrexate (25 doses), with continued tumor growth, followed by sorafenib, which was discontinued after a short trial of therapy as the patient tolerated the medication poorly and developed a severe widespread dermatitis requiring hospitalization. The patient’s tumor continued to grow, measuring up to 21.4 × 8.3 × 7.1 cm, approximately 770 cm^3^, prior to treatment.

**Fig. 1:**
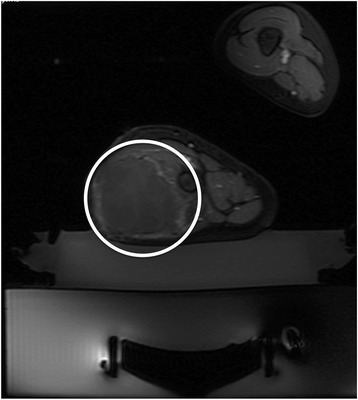
Axial spoiled gradient echo fat-saturated postcontrast image immediately following the first MRgFUS treatment in a 7-year-old boy with large desmoid tumor within the musculature of the thigh. Near total non-perfused volume (*white circle*) is seen at this level within the central portion of the mass, with minimal rim enhancement

MRgFUS of the tumor within the thigh was performed under general anesthesia. The patient was positioned in the right lateral decubitus position on a 3.0T system (Discovery MR750w, GE Medical Systems, Milwaukee, WI). MRgFUS was performed with the ExAblate 2000 MRgFUS system with an in-table 1-MHz transducer (InSightec, Tirat-Carmel, Israel). The procedure was performed with 96 treatment sonications. Total energy per sonication ranged between 1423 and 2846 J with an average of 2168 J. Each individual sonication was 20 s long. Temperature varied between 60 and 85 °C with each sonication. Postcontrast imaging immediately following the treatment demonstrated 75% non-enhancing volume (Fig. 1).

However, the patient’s first treatment was complicated by a third-degree far-field skin burn. This burn occurred in the region where sound energy was leaving the patient, as opposed to the near-field skin entry site which was submerged in a small water bath. On average, the center of the typical sonication spot was approximately 3–4 cm away from the region of the burn. The overall region of the burn measured up to 17 × 10 cm. The parents deferred skin grafting, and the burn was treated with silvadene cream. The area healed within four months following the initial treatment.

Because of the significant non-perfused volume following the first treatment and the potential side effects of alternative therapies, the parents and referring clinicians referred the patient for additional MRgFUS therapy. Three enhanced safety measures were developed to reduce the likelihood of a burn.

First, MR-compatible fiberoptic probes (Lumasense Inc, Santa Clara, CA) were used to directly monitor skin temperature where the far-field beam exited the patient. A small amount of ultrasound gel was placed between the skin and the probe. Temperatures that measured pretreatment were typically around 30–31 °C. A temperature rise of 2–3 °C was noted at the peak of each sonication, with a temperature return to approximately 0.2 to 0.5 °C above the baseline temperature by the end of the cooling period. Theoretically, there is a small risk of heating between the probe and the skin; however, we did not see clinical evidence of significant heating in this region.

In addition to the fiberoptic probes, cold plastic water bags were placed along the far-field skin in order to both directly cool the skin and serve as a conduit for acoustic energy to propagate beyond the far-field air-skin interface. The plastic water bags were refrigerated to approximately 1.6 °C the night before the procedure. No active cooling mechanism was used to maintain their temperature during the procedure. Of note, the use of water bags resulted in a large signal void centrally within the MR image by confounding the scanner’s autoprescan function, resulting in overflipping of the spins. This artifact was corrected by decreasing the transmitter gain by about 20% in manual prescan.

Finally, in addition to the probes and water bags, treatment team members developed a system of regular skin checks every 15–20 sonications in order to directly monitor any changes in the skin.

The patient went on to have four subsequent treatments in the 14 months since the first treatment (Table 1). While tumor regrowth occurred between treatments, each successive treatment resulted in progressively increased non-perfused volume of the tumor, which measured up to 90% following the patient’s most recent treatment. Single small subcentimeter blisters were observed in the far-field following two of these subsequent treatments, which may represent a small area where air was trapped since the water bags did not perfectly conform to the contour of the patient’s skin. Otherwise, no complication was observed, and these blisters resolved within the first week following the treatment.

**Table 1:** Viable and total tumor volume in the months prior to and then following several MRgFUS treatments of a large desmoid tumor in the posterior thigh of a 7-year-old boy

Time	Total tumor volume (cm^3^)	Viable tumor volume (cm^3^)
−5 months	591.8	591.8
−2 months	724.4	724.4
0 months (after first treatment)	771.7	351.6
1 month	704.9	184.7
6 months (after second treatment)	431.9	204.1
8 months	406.8	160.1
10 months (after third treatment)	380.4	197.8
14 months (after fourth treatment)	481.8	70.6 (14.7%)

Following the most recent treatment after 14 months, there was only 14.7% viable tumor volume, and the overall tumor size had decreased significantly from the prior treatment

### Case 2

A 21-year-old woman with a history of a biopsy-proven sporadic desmoid tumor within the posterior left thigh was referred for MRgFUS therapy. The patient had previously been treated with sulindac and celexocib therapy; however, she palpated interval enlargement and increased firmness of the mass while taking these medications. A repeat MRI was performed demonstrating interval growth of the mass which measured up to 13.5 cm in maximum dimension, increased from 11.5 cm 1 year prior. Referral was made to radiation oncology. However, there was concern for significant left ovarian radiation exposure and risk of infertility. Referral was also made to orthopedic oncology. However, the patient was advised that resection would be associated with a significant risk of recurrence and damage to the adjacent neurovascular structures within the posterior thigh, including the sciatic nerve.

At this time, the patient was referred for MRgFUS therapy. The tumor measured up to 24 × 8.6 × 8.5 cm in maximum dimension with pretreatment volume of approximately 730 cm^3^ and was associated with medial displacement of the sciatic nerve. Her symptoms at this time included limiting tolerance for sitting and difficulty with strenuous activities that relied on use of the left hamstring group.

MRgFUS of the tumor within the thigh was performed under general anesthesia. The patient was positioned in the left lateral decubitus position in order to minimize the risk to the sciatic nerve. MRgFUS was performed with the same MRgFUS system. Because despite the rapid growth of her tumor, the patient was relatively high-functioning; only the central portion of the tumor was targeted. The procedure was performed with 38 treatment sonications. Enhanced safety measures were used as described in case 1. Total energy per sonication ranged between 1062 and 1911 J with an average of 1539 J. Each individual sonication was 20 s long. Temperature varied between 52 and 77 °C with each sonication. Thermal dose volume was 25.7 cm^3^. Postcontrast imaging immediately following the treatment demonstrated a relatively low non-perfused volume of the central half of the mass, which measured approximately 30% of the target or 15% of the entire mass (Table 2). Review of the sonication data revealed that 20/39 treatment sonications did not definitely reach 60 °C, including 10/39 which did not clearly reach 57 °C based on the MR thermometry sequences. Overall, the average temperature was 60.8 °C following each sonication. Review of the imaging data demonstrated a subtle fascial band in the soft tissues which potentially contributed to poor heating during the treatment. Several sonications did demonstrate heating accumulating along this interface.

**Table 2:** Viable and total tumor volume in the months prior to and then following several MRgFUS treatments of a large desmoid tumor in the posterior thigh of a 21-year-old woman

Time	Total tumor volume (cm^3^)	Viable tumor volume (cm^3^)
−20 months	454.0	454.0
−2 months	730.7	730.7
0 months (after first treatment)	803.7	694.1
4 months	690.0	678.8
7 months (after second treatment)	789.2	549.6

Treatment effect was greater in the second treatment compared to the first

**Fig. 2:**
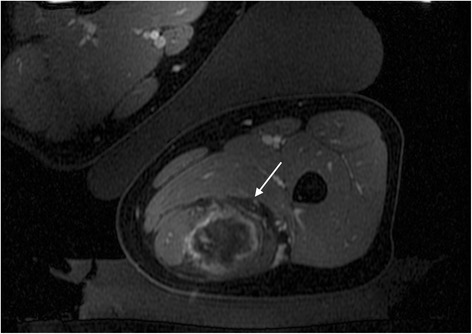
Axial spoiled gradient echo fat-saturated postcontrast image immediately following a second MRgFUS treatment in a 21-year-old woman with desmoid tumor in the posterior compartment of the thigh. Near total non-perfused volume is seen at this level despite close proximity to the sciatic nerve (*white arrow*), with minimal rim enhancement

The patient returned after 6 months for a repeat treatment. The upper half of the tumor was targeted. A slight obliquity of the left lateral decubitus position was used in order to shift the location of the previously noted fascial band. The procedure was performed with the same treatment setup, and 117 treatment sonications were performed. Because the procedure tolerated the first procedure well without any evidence of a skin injury, significantly increased energy was also used compared to the prior treatment with the energy per sonication ranging between 1474 and 6026 J with an average of 3861 J. Thermal dose volume measured 74.3 cm^3^. There was approximately 70% non-enhancing volume within the targeted superior 1/2 of the mass (where there had been the most interval growth following the prior treatment) (Fig. 2). Interestingly, despite the increased non-perfused volume, average temperature was slightly lower in this treatment with an average of 59.0 following each sonication. The patient experienced no complications following either treatment.

### Case 3

**Fig. 3:**
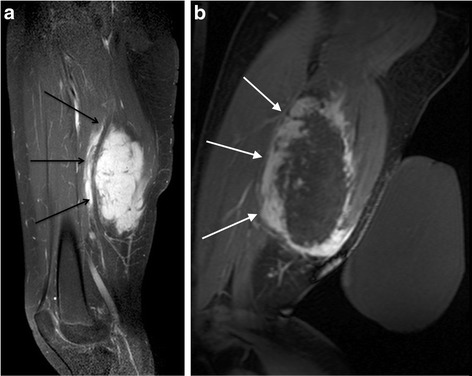
Sagittal spoiled gradient echo fat-saturated postcontrast images **a** before and **b** immediately following MRgFUS treatment in a 14-year-old girl. The pretreatment image clearly demonstrates the sciatic nerve coursing through the posterior aspect of the mass (*black arrows*). Post-treatment plane demonstrates large non-perfused volume with relative sparing of the region immediately adjacent to the nerve (*white arrows*)

The third patient was a 13-year-old girl with a biopsy-proven sporadic desmoid tumor within the posterior aspect of the left thigh. The lesion was initially discovered by palpation by the patient’s parents, with a rapid increase in size over the subsequent 2 months. Pretreatment tumor volume was approximately 440 cm^3^ with dimensions of 12.9 × 8.7 × 5.9 cm. The anterior portion of the tumor circumferentially encased the sciatic nerve over 9 cm of the length of the nerve. The left lateral decubitus position and enhanced safety measures were again used as described previously. Sixty-five treatment sonications were used which ranged in energy between 1790 and 2268 J with an average of 1952 J. Because of the close relationship with the sciatic nerve, relatively low energies were used during the first treatment. Thermal dose volume measured 23.9 cm^3^. There was a relatively low non-perfused volume of approximately 30% (Table 3). The patient returned after 7 months for repeat treatment. The procedure was performed with the same treatment setup, and 140 treatment sonications were performed. Because the procedure tolerated the first procedure well without any evidence of a skin injury, significantly increased energy was also used compared to the prior treatment with the energy per sonication ranging between 1502 and 6094 with an average of 3362 J. These increased energies resulted in an increased non-perfused volume of approximately 70% (Fig. 3). The patient experienced no complications following either treatment.

**Table 3:** Viable and total tumor volume in the months prior to and then following several MRgFUS treatments of a large desmoid tumor in the posterior thigh of a 21-year-old woman

Time	Total tumor volume (cm^3^)	Viable tumor volume (cm^3^)
−3 months	321.8	321.8
0 months (after the first treatment)	381.5	259
3 months	400.7	400.7
6 months (after second treatment)	424.8	121.9

Treatment effect was greater in the second treatment compared to the first

### Discussion

MRgFUS of desmoid tumors is a promising new indication for these difficult to treat, locally aggressive neoplasms. Treatments of these tumors are safe and effective, with a recent retrospective study of 15 patients demonstrating median viable target tumor volume decreasing 63% and pain improved to 2.7 from 7.5, on a 1 to 10 Numerical Rating Scale. Skin burn was the most common complication [5].

However, performing treatments within the thigh can be challenging, as is the case for other local therapies. The first case underscores the importance of effective skin protection in focused ultrasound treatments of the extremities. While the near-field skin is generally well-protected by means of a shallow cool water bath within the table overlying the transducer, the far-field skin is also at risk. Technically, far-field heating can be difficult to observe: a single sonication that successfully results in heating of the target within the desmoid will only allow minimal sound energy to propagate through toward the far-field skin. However, there is significant reflection at the skin-air interface and in large volume sonications, as is performed for many desmoid tumors; the cumulative effect can result in significant heating of the far-field skin. Of note, because this heating is confined to the very superficial interface composed of a thin dermal layer, it is very difficult to observe with MR thermometry or changes in subcutaneous signal intensity. We have found that the most reliable means of directly observing the heating is the placement of a fiberoptic temperature probe along the far-field skin, which provides real time feedback during the course of the sonication, in addition to direct observation of the skin at periodic intervals during the course of treatment.

In terms of skin protection, the use of an acoustic conduit is essential to protect the far-field skin for large volume treatments. Strategies include positioning multiple water bags and gel pads, which can be pooled before the treatment, along the far-field skin to provide more direct thermal protection. Water bags offer the additional advantage of transparency, allowing for direct visualization of the skin without significant alterations to the treatment setup.

The second two treatments also highlight the importance of energy selection for desmoid tumors. The major focused ultrasound systems used to treat desmoid tumors currently rely on software parameters tailored for treatment of uterine fibroids, and while these tumors share certain fibrosis promoting features in common, differences in vascularity and specific monoclonal proliferation (smooth muscle cells in the case of fibroids versus fibroblasts in the case of desmoids) contribute to differential acoustic properties which might require unique treatment parameters. Future studies aimed at optimizing treatment parameters for different tumor types will be important to improving treatment plans and patient outcomes.

## Conclusions

In summary, MRgFUS is an effective treatment for desmoid tumors within the thigh. Large volume treatments near critical structures are feasible, offering an important advantage compared to other local therapies.

## Abbreviations

MRgFUS: Magnetic resonance-guided focused ultrasound
MRI: Magnetic resonance imaging
